# Ergot in Retention of Urine

**Published:** 1853-11

**Authors:** 

**Affiliations:** Lyons


### ECLECTIC DEPARTMENT.

### *On Ergot of Rye in Retention of Urine.*

BY M. PASSOT, OF LYONS.

Ergot of rye has not only the property of exciting the uterine contractions in cases of inactivity of the uterus, but is also very efficacious in the retention of the urine which is caused by atony and paralysis of the bladder. MM. Baudin and Payan were the first who endeavored to demonstrate that this agent does not act on the uterus alone, but rather on the lower part of the spinal cords. They also speak very highly of it as well in the affection which we have now under consideration, as in weakness or paralysis of the lower extremeties.

Drs. Kinsley, Canuto-Canuti, Sainmont and Allier have also recorded cases which bear favorabie testimony to the utility of ergot in paralysis of the bladder. I will now briefly mention some of them:

Capt. B., aged 60, suffered from dysuria, which had increased greatly during the last three months, until it suddenly turned to a complete retention, which necessitated the employment of the catheter several times a day. For two months a host of remedies were used without avail; there was not the slightest improvement. The prostate became enlarged, and the patient suffered much from the use of the catheter, which had to be passed twice every day.

About eight grains of ergot of rye, infused in a cupful of boiling water, was administered three times a day. At the expiration of six hours, the patient passed a small quantity of uiiue, and required the use of the catheter only once in the day. Afterwards it was only passed once in the forty-eight hours, and after ten days the bladder was left to itself.

A lady, aged about 75, was affected with paralysis of the bladder, which for a time required the use of the catheter. Ergot was prescribed in doses of about twenty-eight graius in infusion. On the sixth day of this treatment, it was no longer necessary to pass the instrument, the patient being able to pass water spontaneously.

A man, named Rousseau, aged 58, of a nervous temperament, was, in a fit of passion, attacked with a complete inabilaty to urinate. The bladder was obliged to be emptied by the catheter. The inertia of this organ continued in spite of cold injections to it, cold enemata, the application of ice, also a blister to the hypogastrium.

Strychnine applied, by means of ointment, as a dressing to the blister, also by frictions in the axillæ, on the following day produced cramps in the legs and arms, and was presently accompanied by stiffness, so that it became necessary to discontinue the use of this remedy. There was not the slightest action on the bladder. It was at this stage that the author conceived the idea of giving ergot of rye. He prescribed about ninety-two grains coarsely powdered, to be put into about thirty-four ounces of water, macerate for two days, filtered, and injected cold into the bladder. Seven minutes afterwards the patient experienced a desire to urinate, which, howevor, he could not then satisfy. The next morning the injection was again administered. Eight minutes after he had vesical tenesmus, and then spontaneous emission of urine. The injections were continued for some days. The cure was complete.

In 1848, Dr. Allier of Mareigny, sent a letter to the National Academy of Medicine, in which he gives as the result of his observations that in only one out of fourteen cases ergot proved of no use.

I also am in possession of some cases which, in an incontestible manner, prove that ergot is capable of restoring the contractility of the bladder. The following is the most remarkable:—In the month of July, 1846, I was consulted by M. H., aged 60, of a dry constitution, and a very well-marked nervous temperament. Mr. H, admits having indulged in venereal excesses, and in the excess of the table, and it is these that he blames for the vesical paralysis from which he is now suffering, and which requires the catheter twice a day: otherwise there is no symptom of organic alterat on, no fever, no enlarged prostate. The canal of the urethra is free, through its entire length, and the urine when drawn off is perfectly clear. After having experienced the uselessness of tincture of cantharides and blistering the hypogastrium, I used the following prescription:

| | | |
|---|---|---|
| Freshly powdered ergot, | - | 40 grains, |
| Mucilage, | - - - - | 4 ounces. |

A tablespoonful every half hour.

| | | |
|---|---|---|
| Ergot of rye, powdered, | - | 30 grains, |
| Cocoa butter, | - - - | A sufficiency. |

To be made into two suppositories; one of them to be introduced night and morning.

On the same day at the expiration of some hours, M. H. felt a desire to micturate. At my evening visit I ordered a bath. The patient was scarcely in it before micturation took place spontaneously and with force. From this time to his death, M. H. has always passed water freely and without the assistance of the instrument. I should add, that to make certain of the cure, I continued the remedy for three or four days, but in a decreasing dose. M. H. died on the 30th of January. 1848, of an acute pleuro-pneumonia, during the course of which not a single morbid symptom appeared in the bladder. But with regard to paralysis consecutive to apoplexy, or depending on other affections of the nervous centres, it is well known that they are unaffected by the remedy we are treating of.—*Medical Times and Gazette.*, from French Medical Journals.

